# A Mixed-Methods Examination of Factors Related to HPV Vaccination Promotion in Private Dental Settings, Iowa, 2019

**DOI:** 10.5888/pcd18.200553

**Published:** 2021-03-25

**Authors:** Natoshia Askelson, Grace Ryan, Susan McKernan, Aaron Scherer, Eliza Daly, Lejla Avdic

**Affiliations:** 1 College of Public Health, University of Iowa, Iowa City, Iowa; 2 Public Policy Center, University of Iowa, Iowa City, Iowa; 3 College of Dentistry, University of Iowa, Iowa City, Iowa; 4 College of Medicine, University of Iowa, Iowa City, Iowa

## Abstract

**Introduction:**

Human papillomavirus (HPV)-associated oropharyngeal cancer rates are rising, particularly in males, although rates of other HPV-related cancers are decreasing. Although the HPV vaccine is safe and effective, vaccination rates remain below the Healthy People 2030 goal of 80% coverage. Engaging dental providers, who have experience with patient education and oropharyngeal cancer, may prove useful in efforts to increase vaccination rates. Our research explores dental providers’ (dentists, dental hygienists) willingness to participate in continuing education about HPV, educate parents of adolescents, recommend the vaccine for adolescents, and refer parents to medical providers.

**Methods:**

We used a mixed-methods approach and conducted a survey with dental hygienists and semistructured interviews with dental providers. We produced frequencies and descriptive statistics for all variables and used regression modeling to explore factors related to willingness to promote the HPV vaccine. We used a deductive approach to code interview transcripts.

**Results:**

Regression models using survey data (n = 470) showed that after controlling for demographic and practice-level characteristics, higher levels of willingness were associated with thinking that parents would act on a recommendation and thinking that engaging in HPV promotion is within the scope of practice. Interview data reflected willingness of dental providers to work on HPV vaccination, but revealed barriers (eg, time, knowledge) that need to be addressed.

**Discussion:**

Overall, dental providers expressed a willingness to participate in HPV vaccine promotion, and future efforts should focus on addressing barriers to doing so. Engaging dental providers in HPV vaccine recommendation and referral can help prevent future HPV-related cancers.

**Summary**

**What is already known on this topic?**

Research has shown that dental providers are willing to be involved in HPV vaccine promotion, but little is known about factors related to that willingness.

**What is added by this report?**

Our research identified important factors to address in future programs to help dental providers in HPV vaccine promotion. Our results aid design for an intervention to educate parents, to recommend the vaccine, and to refer parents to local providers.

**What are the implications for public health practice?**

Engaging dental providers in HPV vaccine promotion will give parents another source of information on the vaccine and ultimately help to increase vaccinations and reduce oropharyngeal cancers.

## Introduction

Human papillomavirus (HPV) causes an estimated 34,800 cases of cancer annually in the United States (1). Although rates of HPV-associated cervical cancers have fallen, HPV-associated oropharyngeal cancer rates are increasing. Males are disproportionately affected, experiencing an increased rate of 2.7% per year between 1999 and 2015 (2). In 2020, the Food and Drug Administration (FDA) expanded its approval of the HPV vaccine to include prevention of HPV-related oropharyngeal cancer (3). The HPV vaccine prevents multiple types of cancer; however, uptake falls short compared with other adolescent vaccines, particularly for males (4), and rates remain below the Healthy People 2030 goal of 80% completion (5).

The increase in HPV-associated oropharyngeal cancer and little progress in vaccination rates raises questions about current approaches to vaccine promotion and highlights the need to seek new partners. Dental hygienists and dentists (collectively called dental providers) have been identified as a group to enlist in HPV vaccine promotion (6,7). The American Dental Association (ADA) released a statement that *“*urges dentists, as well as local and state dental societies, to support the use and administration of the HPV vaccine” (8). Moreover, dental providers have long been essential in providing preventive health care, with documented success helping patients understand diabetes management (9) and tobacco cessation (10).

Despite these professional recommendations, most dental providers lack the formal education or experience to ensure that they can be successful partners in vaccine promotion (11,12). We designed our study to expand our understanding of the factors that contribute to dental providers’ willingness to implement routine HPV vaccine promotion. We used a mixed-methods approach to answer the following formative research questions about dental providers in private practice: 1) In what HPV vaccine promotion activities do dental providers currently engage? 2) How willing are dental providers to engage in HPV vaccine promotion? 3) What factors are related to dental providers’ willingness to engage in HPV vaccine promotion?

## Methods

With the goal of using this formative research to inform intervention development, we conducted a mail-based survey of dental hygienists and individual interviews with dentists and dental hygienists. We reported results in this analysis from participants working in a private practice only. Our intention was to develop an intervention focused on dental hygienists; therefore, we conducted both surveys and interviews with that population. We included dentists in the interviews to understand their perception of dental hygienists’ ability to participate in an HPV-focused intervention and their willingness to be supportive of these efforts. Our work was determined not to be human subjects research by the University of Iowa’s institutional review board.

### Mailed survey to dental hygienists

By using existing research (11,13–15), we designed a 31-item questionnaire to assess individual characteristics, HPV-related measures, clinic characteristics, and individual willingness to incorporate HPV vaccine promotion into practice (Table 1). HPV-related measures included current vaccine promotion activities, the extent to which hygienists viewed themselves as vaccine promoters, and their willingness to be involved in vaccine promotion activities. We included items about demographics and personal beliefs, because previous studies found associations among religiosity, political beliefs, and perceptions of the HPV vaccine (16–18). We obtained a list of all licensed dental hygienists in Iowa from the Iowa Dental Board; surveys were mailed to 2,074 dental hygienists in May 2019. We sent a postcard reminder after 10 days to dental hygienists who had not completed the survey, followed by a second survey mailing to all non-completers after 20 days.

**Table 1:** Dental Hygienist Survey Items and Regression Coefficients from Model 3 (N = 470), Iowa, 2019

Variable			Willingness			
			To participate in CE	To educate parents	To recommend vaccine to parents	To refer patients
**Characteristics**	**n (%)**	**Mean (SD)**	**β (SE)**	**β (SE)**	**β (SE)**	**β (SE)**
**Age, y**						
18–26	NA		−.616 (.329)^a^	−.230 (.292)	−.140 (.293)	−.128 (.312)
27–39	1 [Reference]		[Reference]	[Reference]	[Reference]	[Reference]
40–59	NA		−.348 (.169)	−.007 (.150)	−.093 (.150)	−.014 (.160)
≥60	NA		−.064 (.269)	−.008 (.239)	−.124 (.240)	−.063 (.256)
How many years have you been employed as a dental hygienist?	NA	18.64 (11.20)	−.004 (.008)	−.013 (.007)	.002 (.007)	.006 (.007)
How would you describe your political views? (very conservative, 1 to very liberal, 5)	NA	2.66 (0.88)	−.021 (.068)	.002 (.060)	.058 (.061)	.030 (.065)
How religious are you? (not at all, 1 to very religious, 7)	NA	4.92 (1.54)	−.031 (.039)	.009 (.034)	.006 (.034)	.017 (.037)
**Previous experience (yes)**						
Have you or anyone you know been diagnosed with an HPV-related cancer?	77 (16.4)	NA	.106 (.142)	.026 (.126)	.078 (.127)	.134 (.135)
Have you received at least 1 dose of HPV vaccine?	95 (20.4)	NA	−.001 (.151)	.075 (.134)	.255 (.135)	.184 (.144)
Have you completed a continuing education course on either HPV or HPV vaccination?	109 (24.4)	NA	−.028 (.083)	−.073 (.064)	−.104 (.074)	.018 (.079)
Have you completed a continuing education course on oral cancer?	414 (88.5)	NA	−.164 (.343)	−.156 (.153)	−.123 (.154)	−.042 (.164)
**Current behavior (yes)**						
Discuss HPV vaccination as oral cancer prevention with parents of adolescent patients	113 (24.0)	NA	−.291 (.121)	−.128 (.166)	−.216 (.167)	−.268 (.177)
Discuss the HPV vaccine as oral cancer prevention with adolescent patients	97 (20.7)	NA	.396 (.197)^a^	.116 (.175)	.244 (.176)	.247 (.187)
**Attitudes (strongly disagree, 1 to strongly agree, 5)**						
Parents would act on a recommendation from me to vaccinate their adolescent against HPV	NA	2.90 (0.80)	.169 (.072)^a^	.250 (.063)^c^	.244 (.064)^c^	.258 (.068)^c^
It is within a dental hygienist’s scope and role to recommend the HPV vaccine	NA	2.94 (0.95)	.193 (.064)^b^	.333 (.056)^c^	.543 (.057)^c^	.473 (.061)^c^
Local stigma plays a role in discussing HPV and the HPV vaccine in the dental office	NA	3.28 (0.97)	.022 (.057)	−.030 (.051)	−.010 (.051)	.007 (.054)
There are no established professional guidelines pertaining to hygienists recommending the HPV vaccine	NA	3.79 (0.76)	.133 (.073)	.075 (.064)	−.040 (.065)	−.015 (.069)
**Clinic characteristics**						
Accepting new Medicaid patients	101 (21.5)	NA	.017 (.127)	.151 (.113)	.012 (.113)	.214 (.121)
Percent of adolescent patients seen	NA	NA	−.003 (.004)	−.001 (.003)	.000 (.003)	.000 (.004)
Someone hands out info about HPV vaccine to adolescent parents	7 (1.5)	NA	−.119 (.535)	.078 (.474)	.204 (.477)	.308 (.507)
Health history form includes question about HPV	32 (6.8)	NA	−.313 (.220)	−.073 (.195)	−.417 (.196)^a^	−.155 (.208)
Patient waiting area has info about HPV vaccine	10 (2.1)	NA	.029 (.415)	.024 (.368)	−.075 (.369)	.473 (.393)
Dental professionals or staff recommend HPV vaccine to adolescent patients or parents	15 (3.2)	NA	.112 (.309)	.369 (.274)	.316 (.275)	.269 (.293)
**Willingness (very unwilling, 1 to very willing, 5)**						
To educate your patients about HPV and oral cancer, if trained	NA	3.87 (1.00)	NA	NA	NA	NA
To recommend the HPV vaccine to the parents of your adolescent patients	NA	3.43 (1.09)	NA	NA	NA	NA
To refer eligible patients to receive the HPV vaccine with their primary care providers, if trained	NA	3.50 (1.11)	NA	NA	NA	NA
To participate in continuing education about HPV and oral cancer	NA	4.23 (1.03)	NA	NA	NA	NA

Abbreviations: CE, continuing education; HPV, human papillomavirus; NA, not applicable.

a *P* <.05.

b *P* <.01.

c *P* <.001.

We considered 4 primary outcomes related to dental hygienists’ willingness to complete the following vaccine-related activities: 1) Participate in continuing education (CE) about HPV and oral cancer, 2) Educate patients about the HPV vaccine, 3) Recommend the HPV vaccine to parents of adolescent patients, and 4) Refer parents of adolescent patients to eligible providers for HPV vaccination (Table 1). We defined a recommendation for the HPV vaccine as the dental hygienists’ endorsement of the vaccine to a parent of an adolescent patient, whereas a referral is the act of connecting a parent to a local vaccinating provider.

Most items were analyzed by using the original response categories, but, for several questions, we collapsed response categories as a result of small numbers. Discussing the HPV vaccine as oral cancer prevention with either parents or adolescent patients had responses ranging from 1 for never to 5 for always, but we dichotomized responses for those items. Never discussed became 0, and responses 2–5 for the same discussion became 1 for discussed.

Responses to items measuring practice activities, personal HPV vaccination, knowing someone with an HPV-related cancer, or whether the practice accepted Medicaid were dichotomized into 0 for no, unsure, or I don’t know, and 1 for yes.

To explore factors driving dental hygienists’ willingness to participate in HPV vaccine promotion activities, we used a hierarchical model-building approach to generate multiple linear regression models. For each outcome, we considered 3 models. Model 1 included individual demographic characteristics; model 2 added variables related to attitudes and beliefs; and model 3 incorporated practice-level characteristics.

### Interviews with dental hygienists and dentists

We conducted telephone interviews with dental hygienists (March–July 2019) and dentists (July–August 2019) to assess their willingness to perform specific activities related to HPV vaccine promotion and their preferred methods of receiving educational information. Semistructured interview guides were created using questions written by the research team, existing tools, and adapted closed-ended survey items (15,19). We sent the dentist interview guide to a dentist for review and then met as a team to revise questions based on the dentist’s feedback. For both sets of interviews, we used a multipronged sampling strategy that included predetermined sampling frames and active recruitment at area conferences for dental providers.

### Dental hygienists

We stratified dental hygienists who completed the survey by congressional district (n = 4) and randomly selected 25 dental hygienists from each district (n = 100). Invitations were sent to a new set of 100 randomly selected dental hygienists after an inadequate response rate for the first round of invitations (10%). Dental hygienists were sent an initial letter and follow-up postcard inviting them to sign up for an interview online. We also recruited dental hygienists at 2 local dental professional conferences (n = 5).

### Dentists

Our sampling frame for dentists comprised the alumni list (N = 1,642) provided by the University of Iowa College of Dentistry, from which 76% of the state’s dentists graduate (20). We first eliminated anyone from the list without an email address, leaving 905 potential participants. We originally drew a random sample of 300 dentists from this pool of 905 and conducted recruitment via either email or phone. When this failed to yield a satisfactory number of interviews (n = 12), we mailed recruitment materials to the remainder of the sample (n = 605).

All interviewees who completed an interview were sent a $25 gift card for their choice of a local grocery store or gas station. Interviews were audio-recorded and transcribed by a third-party service. We used the same analytic approach for both sets of interviews. We first developed separate codebooks for the dental hygienist and dentist interviews using a primarily deductive method, in which codes were created based on our research questions and interview guides; however, we also allowed coders to add their own codes if they thought it was warranted after discussion with the coding team. Initially, 2 research assistants coded 2 transcripts from each data collection. To establish reliability between the 2 coders, we used a negotiated agreement process in which the 2 coders met with a third member of the research team to discuss any discrepancies and update the codebooks as needed (21). The remaining transcripts were divided equally among the original coders and coded using NVivo 12 software (QSR International).

## Results

### Participants

We received 597 surveys resulting in a 30.4% overall response rate, but 127 of those participants did not work in a private practice, setting our final sample for which we report results for 470 dental hygienists, a response rate of 22.6%. We completed 19 dental hygienist interviews and 20 dentist interviews. Individual demographic information and practice characteristics for both survey and interview participants are reported (Table 2).

**Table 2:** Characteristics of Interview Participants in HPV Vaccination Promotion Study, Iowa, 2019

Characteristics	Dental Hygienist Survey (n = 470)		Dental Hygienist Interviews (n = 19)		Dentist Interviews (n = 20)	
	n/mean	%/SD	n/mean	%/SD	n/mean	%/SD
Male	NA	NA	0	0	12	60
Female	NA	NA	19	100	8	40
**Age, y**						
18–26	14	3.0	0	0	1	5
27–39	176	37.4	3	15.8	8	40
40–59	228	48.5	12	63.2	3	15
≥60	52	11.1	4	21.0	8	40
Hygiene certificate	213	45.3	2	10.5	NA	NA
Associate’s degree	316	67.2	11	57.9	NA	NA
Bachelor’s degree	147	31.3	6	31.6	NA	NA
≥Master’s degree	5	1.1	0	0	NA	NA
Average % adolescent patients	23.85	12.32	23.16	18.92	20.19	9.91

### Current practices and perceived importance of HPV vaccination promotion by dental hygienists

Few hygienists reported that their practices currently incorporated HPV vaccine promotion (Table 1). For these questions, we coded our data so that if a participant reported that at any point they performed the activities in question, we coded their response as a “yes” to the activity. The most commonly performed activities were discussing the HPV vaccine as oral cancer prevention with adolescent patients (20.6% of participants) or their parents (24.0%). Fewer participants reported that their practice included a question about HPV vaccination on their health history form (6.8%), or had HPV informational materials in the waiting room (2.1%) or available to hand to patients (1.5%).

Qualitative data from the interviews also reinforced survey results that dental practices are not currently engaged in HPV vaccine promotion efforts and that HPV vaccination was perceived as a relatively low priority (Table 3). Many dental hygienists thought that the HPV vaccine was important, but potentially not important enough to discuss in a dental setting. Dental hygienists identified their top priorities with adolescent patients as oral hygiene, diet, and brushing habits.

**Table 3:** Themes and Representative Quotes From Interviews With Dental Providers, Iowa, 2019

Theme	Subtheme	Group	Example Quote
Currently discussing HPV vaccination in clinic	NA	Dental hygienist	“I don’t typically think about discussing [it] with my dental patients” (hyg_4.2.2019_EB) “To be honest it's not something we've talked about or addressed at all, but it is something that we're starting to hear more information about, that we're talking about [it] as a[n] office [and] communicating more about. We just have a new dentist in the practice so there's a lot of changes going on, so that hasn't been implemented yet” (hyg_7.12.2019_LA)
		Dentists	“it’s not on my radar” (dentist_7.17_2019_ED2) “I do not know much about the HPV vaccination or what is the recommendation for it” (dentist_8.8.2019_EB1)
Willingness to engage in HPV vaccine promotion	Overall willingness	Dental Hygienist	“You know, I would think likely, or somewhat likely, considering we are considered the health field. . . . So, I mean I would assume likely or somewhat likely” (hyg_3.29.2019_EB) “Well, because we do oral cancer screens on all ages, children and adults, so as we're talking about it I feel like if there's concern or if there [is] talk about the topic of the vaccination that we should just bring up in general, it's something that would be” (hyg_7.12.2019_LA)
	Barriers to participation	Dental Hygienist	”I don’t feel I have enough background information on it” (hyg_4.5.2019_WB) “People aren’t gonna understand or…they’re not gonna see [hygienists] as a provider-type” (hyg_4.1.2019_EB) “Because I realize that it shows up in the mouth and that if the kids are sexually active, that could happen. But I don’t know. I would have to say I’m not comfortable with telling somebody they need to get vaccinated for HPV” (hyg_5.3.2019_FP)
		Dentist	“I know that because a lot of [hygienists] have so much to do during the appointments as well, so sometimes adding one more thing for them to do can be tough for them to adapt to” (dentist_8.8.2019_EB2) “Well, just my ability to answer questions about it, you know, I would probably be less likely to talk about it if I felt like I didn't know a whole lot about it, but a simple educational brochure could take care of that” (dentist_8.27.2019_LA)
	Willingness of parents to act on dental professionals’ recommendations	Dental Hygienist	“My patients would definitely feel very comfortable asking me about recommendations because I have a good rapport with my parents and patients” (hyg_7.12.2019_ED).
		Dentist	“It’s an easy sell. Patients are very concerned about cancer” (dentist_7.19.2019_LA) “Hygienists just have these really nice, established relationships with [their] patients” (dentist_8.9.2019_LA) “I do think that people’s response to this issue is going to be somewhat significantly impacted by the level of relationship and therefore trust that exists” (dentist_8.2.2019_LA3)
	Role of dental professionals in HPV promotion	Dental Hygienist	“I think it's more of a medical [matter]. I was in private practice for almost 30 years, and we never brought that up, or educated the person on it. I think most dentists would think that's for the medical provider” (hyg_3.27.2019_WB) “Well, we educate our patients on overall health. That could definitely be incorporated into that” (hyg_6.10.2019_WB)
		Dentist	“Hygienists probably [have a] smaller role than the dentist” (dentist_7.18.2019_LA) “I hadn’t thought about it a lot until recently, but it should be a bigger role” (Dentist_7.18_2019_LA).

For the most part, dentists echoed dental hygienists’ observations that although HPV is important, it is not a priority to discuss with patients in their practices. Several dentists reported that they did not know much about the vaccine or current guidelines about administering it. Dentists similarly identified their top priorities for adolescent patients as oral hygiene, tooth decay or caries, nutrition, and brushing habits.

Overall, dental hygienists reported being willing to engage in a variety of HPV vaccine promotion strategies, including participation in CE (mean = 4.23, SD = 1.03), educating parents (mean = 3.87, SD = 1.00), recommending the vaccine (mean = 3.43, SD = 1.09), and referring eligible patients to receive the vaccine (mean = 3.50, SD = 1.11 (Table 1). We also explored what factors were related to their willingness to engage in each activity. After controlling for demographic and practice-level characteristics, higher levels of willingness were most associated with thinking that parents would act on a recommendation from their dental hygienist and thinking that engaging in HPV promotion is within the scope of practice for a dental hygienist (Table 1). Because the hierarchical models did not differ significantly, we only report the final model for each activity.

### Interviews

**Dental hygienists**. Results from our interviews helped to contextualize findings from the quantitative analyses. We asked hygienists about their willingness to either educate parents about the HPV vaccine or refer parents to a medical provider to get the vaccine. In general, dental hygienists reported a high willingness to educate parents, but lower willingness to refer parents. Those who reported being willing to educate parents gave several reasons. Several dental hygienists identified this as an important issue because of HPV’s connection with oropharyngeal cancer. Several others reported that they may be willing to educate parents but would need more information and specific training on how to do this.

Dental hygienists who reported being unwilling to refer patients cited various barriers. Several dental hygienists thought that this kind of activity would be outside their scope of practice. Other participants cited issues related to the time available during a visit to discuss the vaccine or feeling uncomfortable discussing it. Only 1 participant reported that the barrier would be their perceived discomfort with the relationship between HPV and sexual activity. Finally, despite these barriers, several dental hygienists reported that they thought parents would act on their recommendation. In many cases, dental hygienists reflected on the good rapport they have established with parents of patients and thought that parents respect them.

**Dentists.** We asked dentists about the possibility of either themselves or dental hygienists participating in the described activities in the workplace. Dentists had mixed opinions about how much of a role hygienists could play in vaccine promotion. Some dentists envisioned that vaccine promotion would either need to be a collaborative effort or that hygienists might have a larger role to play than dentists. Those who thought the dental hygienist could have a critical role in promotion activities cited the fact that dental hygienists often have the most uninterrupted time with patients and are the ones with stronger connections to patients and their parents.

Overall, dentists were willing to have dental hygienists educate parents and refer patients for the vaccine, and to send dental hygienists to CE opportunities related to HPV vaccination and promotion. Only a few dentists expressed that they thought dental hygienists may hesitate because of their own personal beliefs about vaccination. Beyond that, dentists reiterated what dental hygienists reported about needing tools and additional training. Overall, dentists thought that CE opportunities would benefit dental hygienists, that information about HPV and its vaccine was important, and that the topic is relevant to their job duties. Finally, dentists had mixed views about how receptive parents would be to a vaccine recommendation from a dental hygienist; however, the majority expressed confidence that dental hygienists would be able to deliver effective messages.

## Discussion

The recent announcement from the FDA regarding the use of the HPV vaccine to prevent head and neck cancers affirms the need to engage dental providers in vaccine promotion efforts. Similar to recent research, our study found low levels of current activities to promote the HPV vaccine in dental offices (11,12); however, willingness was high among our participants to engage in HPV vaccine efforts. Given previous successes of engaging dental professionals in health promotion work (eg, tobacco cessation [10] and diabetes management [9]), we think that this workforce could help make significant strides in HPV vaccine promotion in the short term and the prevention of HPV-related cancers in the long term.

Several themes emerged to help shed light on how best to work with dental providers on HPV vaccination. First, our interviews with dental providers highlighted the crucial role that dental hygienists may have. Dentists pointed to the fact that, because of the amount of uninterrupted time dental hygienists have with their patients, they often have much stronger relationships with adolescents and their parents than dentists do. These relationships could be leveraged to have dental hygienists provide information and education to this patient population. Not only did dentists convey that dental hygienists who supported their practice would be well-positioned to take on this role, but dentists also did not cite many barriers to this work. Most dentists believed that dental hygienists would be open and willing to participate in HPV vaccine promotion activities; however, one did cite a worry that dental hygienists may not want to participate because of their personal beliefs. Moreover, dental hygienists echoed sentiments that they are willing to participate and reported having a good rapport with patients.

Dentists’ willingness to participate in HPV vaccine–focused activities was echoed in results from dental hygienists. Across the 4 regression models that explored factors related to willingness to participate in different types of HPV vaccine promotion activities, the factors with the largest associations were thinking that discussing the HPV vaccine is within their scope of practice and believing that parents would act on a recommendation to vaccinate their adolescents. The belief that parents would act on their recommendation suggests that, for dental hygienists to engage in these behaviors, there must be high levels of response efficacy — the belief that an action will result in the desired outcome (22). In another study examining providers’ knowledge and beliefs about the HPV vaccine and the role of the dental provider, researchers found that higher levels of knowledge about the vaccine were correlated with a belief that providers’ recommendations to parents to vaccinate their children would be effective (23). Dental providers’ beliefs that parents will be open to their recommendations on the HPV vaccine is an important part of future work in leveraging the role of providers.

Two major strengths exist in our study. The first is using mixed-methods data collection and recruiting both dentists and dental hygienists to participate. The results from our survey and interviews complement each other and give validity to our results. Second, although many studies focused only on collecting data from either dentists or dental hygienists, we have gained important insights into how best to work with dental practices on HPV vaccine promotion efforts overall by recruiting from both groups. Additionally, recognizing the limitations of our study and how they influence our results is important for understanding.

Our study had some limitations. First, our relatively low initial response rates for interview recruitment led us to rely on alternative recruitment methods. Although this was useful in boosting the number of participants, it is possible that it also led to a certain level of response bias. Additionally, we only recruited within Iowa with the goal of developing an intervention for this population; therefore, our results reflect those interviewed and are not generalizable beyond the state or the context. Despite these limitations, however, we believe this work offers key insights into how best to work with private practice dental clinics on HPV vaccine promotion.

Engaging dental providers in the fight against oropharyngeal cancer is not a new idea (24,25); however, risk factors for oropharyngeal cancer have changed dramatically, shifting from tobacco use toward HPV infection (26). In light of the current decline in adolescent vaccinations due to the COVID-19 pandemic (27), finding new ways to promote vaccination within this population is essential. Dental providers’ willingness to be partners in HPV vaccine promotion came across strongly in both surveys and interviews, and our data point to several areas where dentists and dental hygienists can be effective partners. Dental hygienists should be the focus of future training as they have strong relationships with patients and families, reporting high willingness to refer patients and, with proper training, recommend the vaccine. We plan to use these results to design an intervention for private practice settings that will increase these activities in the short term and hopefully support other health care providers in their efforts to vaccinate adolescents and close the gap created by COVID-19. Given the strong evidence that provider recommendation results in higher HPV vaccination rates (28,29), engaging this additional group of providers will continue to increase rates, as parents receive needed information from several sources.

## References

[1] Senkomago V , Henley SJ , Thomas CC , Mix JM , Markowitz LE , Saraiya M . Human papillomavirus-attributable cancers - United States, 2012-2016. MMWR Morb Mortal Wkly Rep 2019;68(33):724–8. 10.15585/mmwr.mm6833a3 31437140 PMC6705893

[2] Van Dyne EA , Henley SJ , Saraiya M , Thomas CC , Markowitz LE , Benard VB . Trends in human papillomavirus-associated cancers - United States, 1999-2015. MMWR Morb Mortal Wkly Rep 2018;67(33):918–24. 10.15585/mmwr.mm6733a2 30138307 PMC6107321

[3] Merck & Co. FDA approves Merck’s Gardasil 9 for the prevention of certain HPV-related head and neck cancers. Kenilworth (NJ); 2020. https://www.merck.com/news/fda-approves-mercks-gardasil-9-for-the-prevention-of-certain-hpv-related-head-and-neck-cancers/. Accessed October 19, 2020.

[4] Elam-Evans LD , Yankey D , Singleton JA , Sterrett N , Markowitz LE , Williams CL , National, regional, state, and selected local area vaccination coverage among adolescents aged 13–17 years - United States, 2019. MMWR Morb Mortal Wkly Rep 2020;69(33):1109–16. 10.15585/mmwr.mm6933a1 32817598 PMC7439984

[5] Office of Disease Prevention and Health Promotion. Increase the proportion of adolescents who get recommended doses of the HPV vaccine. Washington (DC); 2020. https://health.gov/healthypeople/objectives-and-data/browse-objectives/vaccination/increase-proportion-adolescents-who-get-recommended-doses-hpv-vaccine-iid-08. Accessed February 25, 2021.

[6] Harris KL , Tay D , Kaiser D , Praag A , Rutkoski H , Dixon BL , The perspectives, barriers, and willingness of Utah dentists to engage in human papillomavirus (HPV) vaccine practices. Hum Vaccin Immunother 2020;16(2):436–44. 10.1080/21645515.2019.1649550 31361179 PMC7062442

[7] Kline N , Vamos C , Thompson E , Catalanotto F , Petrila J , DeBate R , Are dental providers the next line of HPV-related prevention? Providers’ perceived role and needs. Papillomavirus Res 2018;5:104–8. 10.1016/j.pvr.2018.03.002 29524676 PMC5887011

[8] American Dental Association. ADA policy on HPV vaccination. Chicago (IL); 2018. https://www.ada.org/en/member-center/oral-health-topics/cancer-head-and-neck. Accessed October 19, 2020.

[9] Bruno M . The integration of diet and nutrition lifestyle management strategies into the dental office visit for diabetes risk reduction and management. J Am Dent Assoc 2012;143(12):1320–3. 10.14219/jada.archive.2012.0094 23204087

[10] Davis JM , Ramseier CA , Mattheos N , Schoonheim-Klein M , Compton S , Al-Hazmi N , Education of tobacco use prevention and cessation for dental professionals—a paradigm shift. Int Dent J 2010;60(1):60–72. 20361575

[11] Daley EM , Thompson EL , Vamos CA , Griner SB , Vazquez-Otero C , Best AL , HPV-related knowledge among dentists and dental hygienists. J Cancer Educ 2018;33(4):901–6. 10.1007/s13187-016-1156-5 28039675

[12] Patel S , Koskan A , Spolarich A , Perry M , Flood T . Dental professionals’ knowledge, attitudes, and practice behaviors related to human papillomavirus vaccination. J Public Health Dent 2020;80(1):61–9. 10.1111/jphd.12350 31788802

[13] Kepka D , Rutkoski H , Pappas L , Tay DL , Winkler JR , Dixon B , US oral health students’ willingness to train and administer the HPV vaccine in dental practices. Prev Med Rep 2019;15:100957. 10.1016/j.pmedr.2019.100957 31372330 PMC6661379

[14] Poelman MR , Brand HS , Forouzanfar T , Daley EM , Jager DHJ . Prevention of HPV-related oral cancer by dentists: assessing the opinion of Dutch dental students. J Cancer Educ 2018;33(6):1347–54. 10.1007/s13187-017-1257-9 28741269 PMC6280774

[15] Hosking YP , Cappelli D , Donly K , Redding S . HPV vaccination and the role of the pediatric dentist: survey of graduate program directors. Pediatr Dent 2017;39(5):383–9. 29070161

[16] Kahan DM , Braman D , Cohen GL , Gastil J , Slovic P . Who fears the HPV vaccine, who doesn’t, and why? An experimental study of the mechanisms of cultural cognition. Law Hum Behav 2010;34(6):501–16. 10.1007/s10979-009-9201-0 20076997

[17] Scheffer JA , Cameron CD , McKee S , Hadjiandreou E , Scherer AM . Stereotypes about compassion across the political spectrum. Emotion 2020; Epub ahead of print. 10.1037/emo0000820 32597670

[18] Scherer AM . Public Support for Zika Control Policies. OSF Preprints. 2020. https://osf.io/5zefm

[19] Rutkoski H , Fowler B , Mooney R , Pappas L , Dixon BL , Pinzon LM , Pilot test of survey to assess dental and dental hygiene student human papillomavirus-related oropharyngeal cancer knowledge, perceptions, and clinical practices. J Cancer Educ 2018;33(4):907–14. 10.1007/s13187-017-1165-z 28091963

[20] Iowa Dentist Tracking System. Advisory Committee meeting 2019. Accessed Nov 1, 2020. https://medicine.uiowa.edu/oscep/sites/medicine.uiowa.edu.oscep/files/wysiwyg_uploads/2018%20Dentist%20Book%20Website.pdf

[21] Campbell JL , Quincy C , Osserman J , Pedersen OK . Coding in-depth semistructured interviews: problems of unitization and intercoder reliability and agreement. Sociol Methods Res 2013;42(3):294–320. 10.1177/0049124113500475

[22] Witte K . Putting the fear back into fear appeals: the extended parallel process model. Commun Monogr 1992;59(4):329–49. 10.1080/03637759209376276

[23] Arnell TL , York C , Nadeau A , Donnelly ML , Till L , Zargari P , The role of the dental community in oropharyngeal cancer prevention through HPV vaccine advocacy. J Cancer Educ 2019; Epub ahead of print. 10.1007/s13187-019-01628-w 31728921

[24] Applebaum E , Ruhlen TN , Kronenberg FR , Hayes C , Peters ES . Oral cancer knowledge, attitudes and practices: a survey of dentists and primary care physicians in Massachusetts. J Am Dent Assoc 2009;140(4):461–7. 10.14219/jada.archive.2009.0196 19339536

[25] Carr AB , Ebbert J . Interventions for tobacco cessation in the dental setting. Cochrane Database Syst Rev 2012;(6):CD005084. 10.1002/14651858.CD005084.pub3 22696348 PMC3916957

[26] Fakhry C , Cohen E . The rise of HPV-positive oropharyngeal cancers in the United States. Cancer Prev Res (Phila) 2015;8(1):9–11. 10.1158/1940-6207.CAPR-14-0425 25468833

[27] Gilkey MB , Bednarczyk RA , Gerend MA , Kornides ML , Perkins RB , Saslow D , Getting human papillomavirus vaccination back on track: protecting our national investment in human papillomavirus vaccination in the COVID-19 era. J Adolesc Health 2020;67(5):633–4. 10.1016/j.jadohealth.2020.08.013 32933839 PMC7834295

[28] Brewer NT , Hall ME , Malo TL , Gilkey MB , Quinn B , Lathren C . Announcements versus conversations to improve HPV vaccination coverage: a randomized trial. Pediatrics 2017;139(1):e20161764. 10.1542/peds.2016-1764 27940512 PMC5192091

[29] Rahman M , Laz TH , McGrath CJ , Berenson AB . Provider recommendation mediates the relationship between parental human papillomavirus (HPV) vaccine awareness and HPV vaccine initiation and completion among 13–17-year-old US adolescent children. Clin Pediatr (Phila) 2015;54(4):371–5. 10.1177/0009922814551135 25238779 PMC4366339

